# Serum Uric Acid Increases Risk of Cancer Incidence and Mortality: A Systematic Review and Meta-Analysis

**DOI:** 10.1155/2015/764250

**Published:** 2015-10-04

**Authors:** Shushan Yan, Pengjun Zhang, Wei Xu, Yuqing Liu, Bin Wang, Tao Jiang, Changjiang Hua, Xuan Wang, Donghua Xu, Beicheng Sun

**Affiliations:** ^1^Department of Surgical Oncology, The Eighty-First Hospital of People's Liberation Army, Nanjing 210002, China; ^2^Liver Transplantation Center of the First Affiliated Hospital, Nanjing Medical University, Nanjing 210029, China; ^3^Department of Surgery Emergency, People's Hospital of Rizhao, Rizhao 276800, China; ^4^Center of Computer Network, People's Hospital of Rizhao, Rizhao 276800, China; ^5^Department of Cardiology, People's Hospital of Rizhao, Rizhao 276800, China; ^6^Department of Endocrinology, The Affiliated Hospital of Medical College, Qingdao University, Qingdao 266003, China; ^7^Department of Rheumatism, The First Affiliated Hospital, Nanjing Medical University, Nanjing 210029, China

## Abstract

SUA is a potent antioxidant and thus may play a protective role against cancer. Many epidemiological studies have investigated this hypothesis but provided inconsistent and inconclusive findings. We aimed to precisely elucidate the association between SUA levels and cancer by pooling all available publications. Totally, 5 independent studies with 456,053 subjects and 12 with 632,472 subjects were identified after a comprehensive literature screening from PubMed, Embase, and Web of Science. The pooled RRs showed that individuals with high SUA levels were at an increased risk of total cancer incidence (RR = 1.03, 95% CI 1.01–1.05, *P* = 0.007). Positive association between high SUA levels and total cancer incidence was observed in males but not females (for men: RR = 1.05, 95% CI 1.02–1.08, *P* = 0.002; for women, RR = 1.01, 95% CI 0.98–1.04, *P* = 0.512). Besides, high SUA levels were associated with an elevated risk of total cancer mortality (RR = 1.17, 95% CI 1.04–1.32, *P* = 0.010), particularly in females (RR = 1.25, 95% CI 1.07–1.45, *P* = 0.004). The study suggests that high SUA levels increase the risk of total cancer incidence and mortality. The data do not support the hypothesis of a protective role of SUA in cancer.

## 1. Introduction

Serum uric acid (SUA) is one of the most abundant molecules with antioxidant properties in human blood acting as a free radical scavenger and a chelator of transitional metal ion [1, 2]. However, increased SUA is a highly prevalent condition with controversial health consequences. Emerging data has suggested the causative role of elevated UA levels in cardiovascular, respiratory diseases, renal diseases, and metabolic syndrome [2–4]. Hyperuricemia is a consequence of impaired kidney function and can increase the risk of chronic kidney disease and end-stage renal disease [5]. Low levels of SUA are detrimental to the neurons, while high levels of SUA contribute to inflammation and neuroprotection [6].

Interestingly, it has been hypothesized that SUA may confer protective effects on cancer due to its antioxidant property [7]. However, currently published epidemiological studies on the association between SUA levels and cancer-related incidence and mortality have provided conflicting and inconclusive findings possibly because of different study design, source of controls, sample size, and statistical power. The study by Kuo et al. has suggested that low SUA levels are associated with elevated risk of cancer-related mortality compared with high SUA levels, which implicates a protective role of SUA in cancer [8]. On the contrary, Strasak AM and colleagues have demonstrated that high SUA levels are independently related to increased risk of total cancer mortality [9]. To shed light on understanding the paradoxical role of SUA in the risk of cancer incidence and mortality, we performed this meta-analysis of all currently published studies.

## 2. Materials and Methods

### 2.1. Search Strategy

A comprehensive literature screening from databases of PubMed, Embase, and Web of Science was performed for eligible studies from their inception up to August 12, 2014. We used the following terms: uric acid, serum uric acid, gout, or hyperuricemia; cancer, tumor, or carcinoma; cancer risk, cancer incidence, or cancer mortality. The references of all retrieved studies were also screened for additional papers. There were no language restrictions for literature search.

### 2.2. Inclusion and Exclusion Criteria

Studies were included in our study if they conform to the following inclusion criteria: (1) studies in cohort designs; (2) studies on the relationship between SUA and cancer; (3) studies with data of odds ratio (ORs), relative risks (RRs), or hazard ratios (HRs) with 95% confidence intervals (95% CIs). Studies not related to the risk of cancer incidence and mortality, case reports, reviews, animal studies, and studies with overlapping data were all excluded.

### 2.3. Data Extraction

Data were extracted by two investigators independently. Disagreements were resolved by consensus. Relevant data were as follows: first author, year of publication, origins, study designs, specific sites of cancer, cancer types, sex, age, sample size, baseline time, follow-up duration, adjusted factors, and RRs or HRs or ORs with corresponding 95% CIs for the risk of cancer incidence and mortality. Available RRs or HRs or ORs with 95% CIs were extracted based on the highest SUA levels in each included study.

### 2.4. Statistical Analysis

We evaluated the strength for association between SUA levels and cancer by calculating the pooled RRs with 95% CIs. Cochran's *Q*-statistic test and *I* ^2^ test were performed to estimate the between-study heterogeneity, and *P* < 0.05 and *I* ^2^ > 50% suggested potential heterogeneity across all studies [20, 21]. The fixed-effects model by Mantel-Haenszel method was applied when the between-study heterogeneity was insignificant [22]; otherwise, the random-effects model by DerSimonian and Laird method was used [23]. Stratified analyses by sex and specific sites of cancer were also carried out. Sensitivity analysis was performed to assess the influence of single studies. Begg's funnel plots and Egger's test were adopted to estimate publication bias risk [24, 25]. STATA 12.0 software (StataCorp, College Station, TX, USA) was used for all analyses.

## 3. Results

### 3.1. Literature Search and Characteristics of All Studies

We performed a comprehensive literature search in PubMed, Embase, and Web of Science databases for eligible studies. According to the inclusion criteria, 5 independent studies on cancer incidence with 456,053 subjects [4, 10–12] and 12 on cancer mortality with 632,472 subjects [3, 8, 9, 13–19] were finally included in our study. All included studies were in prospective cohort designs, which were published between 1999 and 2014 (Table 1). Other characteristics including first author, year of publication, sample size, follow-up duration, baseline time, sex, and cancer type were presented in Table 1 at length. The study by Hiatt and Fireman estimated roles of SUA in cancer incidence among males and females, respectively, and thus it was regarded as two independent studies [12]. Based on different gender, two other papers on the association between SUA and cancer mortality were also divided into two individual studies, respectively [3, 18].

### 3.2. Association between SUA and the Risk of Cancer Incidence

**Figure 1:**
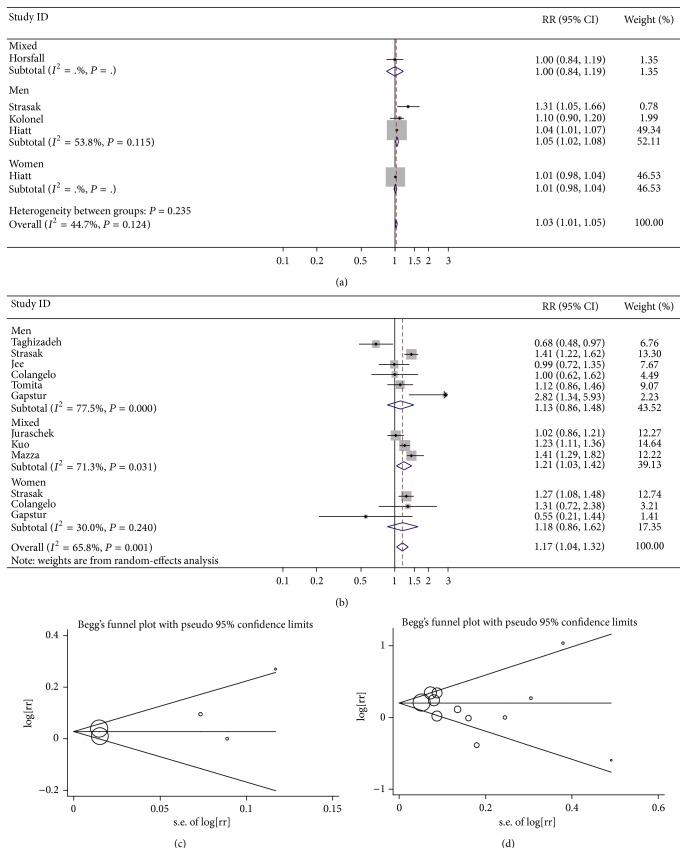
(a) Forest plot for SUA and the risk of cancer incidence; (b) forest plot for SUA and the risk of cancer mortality; (c) Begg's funnel plots for the risk of cancer incidence (*P* value for Egger's test equal to 0.295); (d) Begg's funnel plots for the risk of cancer mortality (*P* value for Egger's test equal to 0.354).

We found that high SUA levels were associated with an increased risk of total cancer incidence by meta-analysis of 5 independent studies (RR = 1.03, 95% CI 1.01–1.05, *P* = 0.007) (Table 2, Figure 1(a)).

When stratifying analysis by sex, the pooled RRs showed that high SUA levels were significantly related to the risk of cancer incidence among males but not females (for men: RR = 1.05, 95% CI 1.02–1.08, *P* = 0.002; for women, RR = 1.01, 95% CI 0.98–1.04, *P* = 0.512) (Table 2, Figure 1(a)). However, there was only one relevant study on the cancer incidence risk in relation to SUA among females.

When stratifying analysis by specific sites of cancer, significant relationship between high SUA levels and the risk of lymphoid and hematopoietic system cancers was observed, but not other specific sites of cancer (Table 2). Sensitivity analysis did not materially alter the pooled results mentioned above.

### 3.3. Association between SUA and the Risk of Cancer Mortality

The pooled RRs revealed that individuals with high SUA levels were at an elevated risk of total cancer mortality (RR = 1.17, 95% CI 1.04–1.32, *P* = 0.010) (Table 2, Figure 1(b)). Such significant association was demonstrated in females rather than males (for females: RR = 1.25, 95% CI 1.07–1.45, *P* = 0.004; for males: RR = 1.13, 95% CI 0.86–1.48, *P* = 0.384) (Table 2, Figure 1(b)). The between-study heterogeneity was not significant in studies performed among women. Thus, fixed-effects model was used to estimate the role of SUA in cancer mortality among females (Table 2). The combined results were further confirmed by sensitivity analysis.

In stratifying analysis by specific sites of cancer, high SUA levels were found to correlate with an elevated risk of digestive cancers mortality (RR = 1.27, 95% CI 1.08–1.49, *P* = 0.003) (Table 2). No significant association was observed in relation to the mortality of other cancers (Table 2). Sensitivity analysis did not modify the pooled results.

### 3.4. Publication Bias

No potential publication bias risk was found in our study, as suggested by both Begg's funnel plots and Egger's test (Figures 1(c) and 1(d)). Similar findings were demonstrated in both studies related to cancer incidence and studies associated with cancer mortality.

## 4. Discussion

The current meta-analysis shows the evidence that high SUA levels are associated with increased risk of cancer incidence and mortality. The association between SUA and the risk of cancer-related incidence and mortality differs by gender. Positive association between high SUA levels and total cancer incidence was observed in males but not females. Besides, high SUA levels were associated with an elevated risk of total cancer mortality in females but not males.

To the best of our knowledge, UA is produced from metabolic conversion of either dietary or endogenous purines. The primary sites of excretion of UA are kidney and gut. UA is capable of scavenging free radical and chelating transitional metal ions by preventing peroxynitrite-induced protein nitrosylation, lipid and protein peroxidation, and the inactivation of tetrahydrobiopterin [2]. Nonetheless, studies have also suggested a prooxidant role of UA [2]. Elevated levels of SUA can enhance inflammatory response and contribute to hyperuricemia, gout, cardiovascular, and renal complications. It is particularly interesting since UA plays dual roles as antioxidant and prooxidant. Concerning malignant diseases, SUA has drawn much attention for the past few decades. High cell turnover and tumor lysis syndrome in certain cancers are responsible for increased levels of UA in human serum, implicating potential link between SUA and cancer [8, 26, 27]. It has been demonstrated that UA can provide an antioxidant defense against oxidant- and radical-caused aging and cancer [7]. Nevertheless, UA has also been reported as an independent risk factor for cancer incidence and mortality in other studies [9, 28]. Thus, the precise relationship of SUA with cancer remains obscure and needs further elucidation.

Tobacco smoking is a well-established risk factor of lung cancer. Horsfall and colleagues have found that current smokers with low levels of SUA are more susceptible to lung cancer, which suggests a protective role of SUA and an interaction between SUA and smoking in lung carcinogenesis [4]. Reversely, the study by Strasak et al. has shown that elevated levels of SUA are related to higher risk of several site-specific malignancies, for instance, digestive organs, connective tissue, soft tissue and skin, lung cancer, urinary organs, bone, and hematopoietic cancers [10]. However, no significant association between increased SUA levels and the risk of total cancer or cancers of lung, stomach, colon, rectum, bladder, or hematopoietic system was demonstrated by Kolonel et al. [11]. The contradictory findings may be attributed to diverse methodology, ethnicity, environmental exposures, source of controls, and statistical power. Meta-analysis with large sample size is more powerful in identifying minor association by pooling all currently available publications. A total of 5 independent studies on cancer incidence and 12 on cancer mortality were included in the present meta-analysis. The pooled results suggested that elevated SUA levels were positively associated with the risk of total cancer and lymphoid and hematopoietic system cancers, implicating a risk role of SUA in carcinogenesis. Moreover, gender bias was observed when estimating the relationship between SUA levels and cancer risk, which warranted elucidation in more relevant cohort studies with large sample size.

The effect of SUA levels on cancer mortality seems inconsistent. Taghizadeh et al. reported that higher levels of SUA were related to lower risk of cancer mortality, which supported a protective role of SUA in the risk of cancer mortality [13]. Nonetheless, elevated SUA levels were demonstrated to be an independent risk factor for cancer-related mortality in several other cohort studies [9, 14, 19]. To shed some light on the controversial findings, we estimated the association between SUA and the risk of cancer mortality by meta-analysis of all currently available data. Statistically significant association was observed between higher SUA levels and increased mortality of total cancer and digestive cancers. Besides, the positive correlation was more significant in females than males. Different amounts of SUA, saturation of UA levels in cancer, and metabolism of SUA may be responsible for the risk discrepancy between women and men with cancer. Interestingly, the significant association was related to digestive cancers, but not other specific sites of cancer, implicating site bias in the relationship between higher SUA levels and cancer mortality. The statistically significant association may be a chance finding in that only one eligible study was included related to the mortality of pancreatic cancer and colorectal cancer, respectively [3, 18]. In addition, the inconsistent findings for diverse specific sites of cancer might be attributed to variable number of years for cancer mortality, different study design, sample size, environmental exposures, and genetic background. More studies with high quality are warranted to provide a precise estimate for the role of SUA in cancer-related mortality, particularly with regard to specific sites of malignancies and gender.

## 5. Limitations

The pooled results should be interpreted with caution due to some limitations in our study. First, significant gender bias was found when assessing the effect of SUA on the risk of cancer incidence and mortality; however, the sample size in each stratified analysis by sex seemed a bit limited. More relevant studies with sufficient statistical power are recommended for further elucidation. Second, a significant interaction between SUA and smoking was demonstrated in the development of cancer [4]. We failed to perform stratified analysis by smoking status for lack of enough available studies published to date. It can be further investigated in more future studies. Last but not least, although no significant publication bias was observed in our study, potential bias might be introduced in that not all included studies were based on the same adjusted factors, such as age, gender, environmental exposures, and ethnicity. Thus, the findings in this meta-analysis should be interpreted seriously.

## 6. Conclusions

In summary, the present study suggests that elevated SUA levels are related to increased risk of cancer incidence and mortality. However, the precise association between SUA levels and cancer warrants further investigation in more independent studies with high quality in the future.

## Figures and Tables

**Table 1:** Characteristics of all studies.

First author	Year	Study design	Origins	Sample size	Followup (years)	Baseline time	Sex	Cancer
Cancer incidence								
Horsfall [4]	2014	Prospective cohort study	UK	205,484	5	2000–2012	Mixed	Lung
Strasak [10]	2009	Prospective cohort study	Austrian	78,850	12.4	1985–2003	Men	All
Kolonel [11]	1994	Prospective cohort study	Hawaii	7,889	27	1965–1968	Men	All
Hiatt [12]	1988	Prospective cohort study	USA	75,283	9.8	1965–1972	Men	All
Hiatt [12]	1988	Prospective cohort study	USA	88,547	9.8	1965–1972	Women	All
Cancer mortality								
Taghizadeh [13]	2014	Prospective cohort study	Dutch	4,350	38	1965–1969	Men	All
Juraschek [14]	2014	Prospective cohort study	Scotland	15,083	22.7	1984–1987	Mixed	All
Kuo [8]	2013	Prospective cohort study	Taiwan	354,110	9	2000–2007	Mixed	All
Strasak [15]	2007	Prospective cohort study	Austrian	28,613	15.2	1985–2005	Women	All
Strasak [15]	2007	Prospective cohort study	Austrian	83,683	13.6	1985–2005	Men	All
Jee [16]	2004	Prospective cohort study	Korean	22,698	9	1992–1996	Men	All
Colangelo [3]	2002	Prospective cohort study	USA	20,433	26.2	1967–1973	Men	Colorectal cancer
Colangelo [3]	2002	Prospective cohort study	USA	15,149	26.2	1967–1973	Women	Colorectal cancer
Tomita [17]	2000	Prospective cohort study	Japanese	49,413	5.4	1975–1982	Men	All
Gapstur [18]	2000	Prospective cohort study	USA	20,475	25	1963–1973	Men	Pancreatic cancer
Gapstur [18]	2000	Prospective cohort study	USA	15,183	25	1963–1973	Women	Pancreatic cancer
Mazza [19]	1999	Prospective cohort study	Italy	3,282	12	Not reported	Mixed	All

**Table 2:** Summary meta-analysis results for the association between SUA levels and cancer.

Group/subgroup	Number	RR (95% CI)	*P* value for pooled analysis	*I* ^2^ (%)	*P* value for heterogeneity analysis
Cancer incidence					
All	456,053	1.03 (1.01–1.05)	0.007	44.7	0.124
Men	162,022	1.05 (1.02–1.08)	0.002	53.8	0.115
Women	88,547	1.01 (0.98–1.04)	0.512	—	—
Specific sites					
Any	250,569	1.03 (1.01–1.05)	0.006	57.9	0.068
Respiratory system and intrathoracic organs	456,053	1.05 (0.93–1.19)	0.448	67.5	0.015
Digestive organs	266,347	1.08 (0.94–1.25)	0.263	63.4	0.018
Urinary organs	86,739	1.17 (0.44–3.15)	0.752	82.3	0.018
Lymphoid and hematopoietic systems	86,739	1.71 (1.10–2.68)	0.018	30.0	0.232
Male genital organs	162,022	1.06 (1.00–1.13)	0.058	56.1	0.103
Cancer mortality					
All	632,472	1.17 (1.04–1.32)	0.010	65.8	0.001
Men	201,052	1.13 (0.86–1.48)	0.384	77.5	<0.001
Women	58,945	1.25 (1.07–1.45)	0.004	30.0	0.240
Specific sites					
Any	561,232	1.17 (1.04–1.32)	0.011	70.7	0.001
Respiratory system and intrathoracic organs	116,646	1.08 (0.61–1.91)	0.786	78.2	0.010
Digestive organs	187,886	1.27 (1.08–1.49)	0.003	40.1	0.124
Urinary organs	112,296	1.35 (0.88–2.07)	0.172	0.0	0.681
Lymphoid and hematopoietic systems	112,296	1.18 (0.82–1.70)	0.364	0.0	0.837
Bone, connective tissue, soft tissue, and skin	112,296	0.94 (0.47–1.87)	0.857	0.0	0.588
Male genital organs	88,033	0.51 (0.07–3.85)	0.516	75.5	0.044

## Abbreviations

ORs: Odds ratios
RRs: Relative risks
HRs: Hazard ratios
95% CIs: 95% confidence intervals
SUA: Serum uric acid.
